# A scoping review of rebel nurse leadership: Descriptions, competences and stimulating/hindering factors

**DOI:** 10.1111/jocn.15765

**Published:** 2021-05-06

**Authors:** Eline de Kok, Anne Marie Weggelaar‐Jansen, Lisette Schoonhoven, Pieterbas Lalleman

**Affiliations:** ^1^ Dutch Nurses’ Association Utrecht Utrecht The Netherlands; ^2^ Julius Center for Health Sciences and Primary Care University Medical Center Utrecht, Utrecht University Utrecht The Netherlands; ^3^ Erasmus School of Health Policy and Management Erasmus University Rotterdam The Netherlands; ^4^ School of Health Sciences, Faculty of Environmental and Life Sciences University of Southampton Southampton United Kingdom; ^5^ HU University of Applied Sciences Utrecht Utrecht The Netherlands

**Keywords:** behaviour, communication, competence, health care, leadership, nurses, quality improvement, review

## Abstract

**Aims:**

To (1) give an overview of rebel nurse leadership by summarising descriptions of positive deviance, tempered radicals and healthcare rebels; (2) examine the competences of nurse rebel leadership; and (3) describe factors that stimulate or hinder the development of rebel nurse leadership.

**Background:**

Research shows nurses have lower intention to leave their jobs when they can control their work practices, show leadership and provide the best care. However, organisational rules and regulations do not always fit the provision of good care, which challenges nurses to show leadership and deviate from the rules and regulations to benefit the patient. Three concepts describe this practice: positive deviance, healthcare rebels and tempered radicals.

**Design:**

Scoping review using the Joanna Briggs Institute methodology and PRISMA‐ScR checklist.

**Methods:**

Papers describing positive deviance, healthcare rebels and tempered radicals in nursing were identified by searching Scopus, CINAHL, PubMed and PsycINFO. After data extraction, these three concepts were analysed to study the content of descriptions and definitions, competences and stimulating and hindering factors.

**Results:**

Of 2705 identified papers, 25 were included. The concept descriptions yielded three aspects: (1) positive deviance approach, (2) unconventional and non‐confirmative behaviour and (3) relevance of networks and relationships. The competences were the ability to: (1) collaborate in/outside the organisation, (2) gain and share expert (evidence‐based) knowledge, (3) critically reflect on working habits/problems in daily care and dare to challenge the status quo and (4) generate ideas to improve care. The factors that stimulate or hinder the development of rebel nurse leadership are as follows: (1) dialogue and reflection, (2) networking conditions and (3) the managers’ role.

**Conclusions:**

Based on our analysis, we summarise the descriptions given of rebel nurse leadership, the mentioned competences and provide an overview of the factors that stimulate or hinder rebel nurse leadership.

**Relevance to clinical practice:**

The descriptions produced in this review of rebel nurse leadership and the stimulating or hindering factors listed should help nurses and managers encourage rebel leadership.

**What does this paper contribute to the wider global clinical community?**

- Organisational rules and regulations do not always fit the provision of good care, which challenges nurses. When nurses show more leadership in daily practice it will influence and enhance the quality of care and help retain nurses.
- The understanding of positive deviants, tempered radicals and healthcare rebels, their competences and the factors that stimulate or hinder the development of rebel nurse leadership will help management and nurses to support and develop rebel nurse leadership in daily practice.

## 1 INTRODUCTION

The increasing demand for nurses (Marć et al., 2019) and their high turnover (Duffield et al., 2014; Fasbender et al., 2019; Li et al., 2018) have resulted in a workforce shortage that has an adverse impact on healthcare quality (Aiken et al., 2018; Ball et al., 2014). Research shows that reasons for nurses to resign include the high workload, job stress and little control over their own professional practice (Aiken et al., 2002; Fasbender et al., 2019; Li et al., 2018). Several studies indicate that nurses have lower intentions to leave their profession if they can control their daily practice and show leadership (Blake et al., 2013; Ducharme et al., 2017; Li et al., 2018). Fully understanding the role of nurse leadership in daily practice is crucial, especially with the current challenge of retaining nurses.

There are many studies on nurse leadership in the literature. These studies often highlight nurse leadership from a leader–follower perspective and resonate with the transformational leadership paradigm (Hutchinson & Jackson, 2013). Transformational leadership focuses on the cultural aspects of an organisation and leaders establishing followers with their vision, norms and belief systems (Hutchinson & Jackson, 2013) that create meaning and motivation for the followers (Bass & Steidlmeier, 1999). Many transformational leadership studies in nursing focus on the hierarchical leader, a designated position of leadership of individuals (e.g. nurse manager and nurse executive) versus healthcare professionals (e.g. nurses) as followers (Reichenpfader et al., 2015; Sfantou et al., 2017; Wong et al., 2013). However, these papers direct ‘little attention […] towards understanding how leadership may be enabled in those not in formally designated leadership positions or how organisational processes can be changed to liberate follower's potential to lead’ (Jackson & Parry, 2011 in Hutchinson & Jackson, 2013, p. 14). Many nurses do not have a designated position of leadership, but any nurse can exhibit leadership in their practices. Clark observes (Clark, 2008, p. 30): ‘Some nurses may not think of themselves as leaders because they equate leadership with authority or with specific job titles rather than as a way of thinking or behaving’ in daily work at the frontline. To understand more about leadership in everyday practices, Leadership‐as‐practice (LAP) theory provides insights into the moral, emotional and relational aspects of leadership in daily working life (Raelin, 2011). Rather than envisioning leadership by its rational, objective and technical aspects (Carroll et al., 2008), LAP helps us understand how leadership is enacted by those not in designated positions. It shines light on how the context influences leadership and the dynamics within organisations that foster leadership. The lens of LAP might provide valuable new insights into nurse leadership in daily practice, how it can be supported and how it could influence the retainment of nurses.

Nurses want to provide the best care for their patients, but they work in organisations with rules and regulations that might not always fit their norms and beliefs on what the best care is. In terms of LAP theory, rules and regulations influence the moral, emotional and ultimately relational aspects of leadership in daily practice (Raelin, 2011). Wallenburg et al. (2019) found that nurses may find it challenging to comply with the organisation's rules and regulations and sometimes also feel that the professional guidelines hinder the provision of best quality care for the individual patient. Gabbay and Le May (2016) state that if professionals want to make a good clinical decision for their patients’ care the variability of the multifarious considerations becomes part of their clinical decisions. The authors stated that no theoretical, research‐based knowledge or clinical guideline could ever be expected to cover all these considerations (Gabbay & Le May, 2016). Therefore, nurses sometimes deviate from the professional norms and organisational rules and regulations to generate better outcomes for their patients or to improve processes on their wards. However, hierarchical leaders do not always permit deviation, which requires individual nurses to show leadership as they must balance between conformity and compliance in order to be a ‘good’ employee and deviation to benefit their patients and the organisation of their wards (Berwick et al., 2017). The literature also describes ‘bad rebelism’ and ‘wrong deviation’ (Bevan, 2010); deviating in your own interest and breaking the rules out of anger only undermines the quality of care (NHS, 2016, slide 66).

Several studies describe professionals showing leadership in daily practice as ‘positive deviance’. Gary defines positive deviance as ‘an intentional and honorable behavior that departs or differs from an established norm; contains elements of innovation, creativity, adaptability, or a combination thereof; and involves risk for the person deviating’ (Gary, 2013, p. 29). Bevan's description of deviating professionals in health care (Bevan, 2010) formed the basis of the online School for Health and Care Radicals, established in 2014, nowadays called the School for Change Agents. The purpose of the school was ‘to develop effective change agents, ultimately contributing to fast, large‐scale, sustainable improvement in health and social care, leading to better patient outcomes’ (Grifford et al., 2015). Bevan defines ‘healthcare rebels’ as ‘committed to the patient‐centred mission and values’ of their organisation and see ‘many possibilities for doing things in different ways’ (Bevan, 2013). The set‐up of the school was inspired by Meyerson's book explaining her research on tempered radicals, individuals who ‘navigate the often murky organisational waters to pursue their ideals while fitting in enough to succeed’ (Meyerson, 2008, p. 8). In addition, several other studies in health care describe professionals showing leadership in daily practice as ‘positive deviants’. The concepts of positive deviance, healthcare rebels and tempered radicals describe nurses who deviate creatively from formal rules and regulations, not in their own interests, but for better health care (quality). Wallenburg et al. (2019) observe that deviating healthcare professionals—nurses—tend to ‘stay under the radar’ of management to achieve their goal of improved patient care. To deviate and find another, better way demands experimentation, trying things out and evaluating the results (Clancy, 2010; Meyerson, 2008; Wallenburg et al., 2019). Given that positive deviants, healthcare rebels and tempered radicals ‘stay under the radar’, it is not surprising that these concepts are seldom mentioned in the nursing leadership literature. However, if rebel nurse leadership is better understood, it might be possible to study this more closely in nursing practice. Therefore, this scoping review provides an overview of perspectives on nurse rebel leadership based on the literature on positive deviance, healthcare rebels and tempered radicals.

## 2 AIMS

In this study, we aim to (1) give an overview of the concepts and descriptions of positive deviants, tempered radicals and healthcare rebels in nursing, (2) examine the competences of rebel nurse leaders, (3) describe factors that stimulate or hinder the development of rebel nurse leadership, resulting in (4) a description of the concept of rebel nurse leadership.

## 3 METHODS

### 3.1 Literature search

A scoping review is a method which provides a preliminary assessment of the potential size and scope of available research literature to identify the nature and extent of research evidence (Grant & Booth, 2009). In conducting our scoping review, we used the Joanna Briggs Institute (JBI) Reviewers’ manual (Peters et al., 2017) and the PRISMA Extension for Scoping Reviews (PRISMA‐ScR) checklist (Tricco et al., 2018; File S1).

First, we undertook a limited search to identify relevant keywords and synonyms to develop an a priori search protocol with a set of inclusion and exclusion criteria. We included three concepts: positive deviance, healthcare rebels and tempered radicals. Vigilantes and Mavericks were excluded, because the definitions and descriptions given in the papers did not match the positive deviating professionals we were aiming for, based on this limited search.

Second, we searched for all the identified keywords and index terms in four databases: Scopus, CINAHL (Cumulative Index to Nursing and Allied Health Literature), PubMed and PsycINFO. The keywords used in the search strings included the following: ‘Rebel*’, ‘Tempered Radical*’, ‘Positive Deviance*’ and ‘Health*’ (see also Appendix S1). One researcher (EdK) developed the search strings, and the whole research team checked and discussed them. The search period ranged from 1 January 1995 (first publication on tempered radicals by Meyerson and Scully [1995]) to 1 April 2020.

Third, we selected additional papers from the reference lists of the included papers. Relevant papers were checked to identify any research specifically on the three concepts (positive deviance, healthcare rebels and tempered radicals) that matched the eligibility criteria.

### 3.2 Review process and data extraction

One researcher (EdK) screened the titles and abstracts of the retrieved papers. Then, two other researchers (PL or AW) independently reviewed a randomly selected sample of ten titles and abstracts. The Fleiss Kappa measure of inter‐rated reliability resulted in 1.0. Inclusion criteria were primary research papers written in English, methodology papers, discussion papers and reviews focusing on nurses or nursing practice in all healthcare sectors, including all patient or disease groups. Exclusion criteria were poster presentations, books, policy papers and interviews with researchers about their research.

Next, the three researchers independently read and assessed the full papers. Any disagreements on assessment were discussed by the research team up to consensus. Papers were excluded if their focus was on the related deviant behaviour or rebelism of patients and if healthcare professional teams or healthcare professionals were discussed in general. Papers focusing on organisational structures and not on the professionals were also excluded. Of the included papers, the literature references were checked, and additional papers were added.

Using a sheet developed by the research team to standardise the data extraction process, one researcher extracted details from the selected papers: author(s), year of publication, country, record type, research aim, study participants, methods, findings related to the aim of the scoping review (descriptions, competences and factors stimulating or hindering the development of rebel nurse leadership) and conclusions. Another researcher checked the extractions. Then, working together, all four researchers sorted the extracted data and accompanying narratives into a form that reflects the aims of this scoping review (see Table 1).

**TABLE 1:** Key competences of included papers

	Title paper, author(s), year	Country	Research aim	Study participants	Methods	Description	Competences	Stimulating/hindering factors	Study conclusions
1	Combating infections at Maine Medical Center: Insights into complexity‐informed leadership from positive deviance (Lindberg & Schneider, 2013)	USA	To study organisational change process known as positive deviance (PD) which sheds light on leadership in a complex organisational context	*N* = 3 pilot inpatient nursing units (oncology, nephrology and dialysis service) at Maine Medical Center in Portland, and a tertiary care centre for northern New England	Exploratory case study Open‐ended, reflexive observation and a grounded theory approach	X	X	X	Non‐managerial employees now have a louder and stronger voice and management does listen to them. But there is an underlying acceptance that while managers might not dominate the conversation as much as they did in the past, their words might matter more than those who have only recently found their voice. All have a voice, but all voices are not equal
2	Beyond the hospital infection control guidelines: A qualitative study using positive deviance to characterize gray areas and to achieve efficacy and clarity in the prevention of healthcare‐associated infections (Gesser‐Edelsburg et al., 2018)	Israel	To study the gray areas in the care continuum in ICUs where systematic guidelines are adhered to only partially by the staff, and where there are no practices of PD individuals that address these gray areas as reported by the staff	*N* = 82 participants at Hadassah Hospital from the GICU and MICU (*N* = 47 nurses, *N* = 14 physicians, *N* = 5 nursing aides, *N* = 5 nursing students, *N* = 2 social workers, *N* = 2 physical therapists, *N* = 1 respiratory technician, *N* = 2 secretaries, *N* = 1 national service volunteer, *N* = 3 cleaning staff	Qualitative constructivist research method. Interviews, observations and video recordings of identified positive behavioural practices	X	X	X	The study characterised the gray areas in the care continuum identified by staff, where solutions were found through PD practices. Instead of investing in producing additional, specific guidelines for different situations and developing training programmes to implement them, it is important to encourage hospital personnel to create their own solutions for different situations on the care continuum, and to disseminate them in the units to achieve a bottom‐to‐top change
3	Methicillin‐resistant Staphylococcus aureus (MRSA) prevention through facility‐wide culture change (Bonuel et al., 2009)	USA	To study one hospital's fight against methicillin‐resistant *Staphylococcus aureus* by implementing a facility‐wide program aimed at changing and standardizing the hospital culture	N.A.	N.A.	X		X	1 year after implementing our best practices and the MRSA bundle in all our 15‐inpatient nursing units, we have 4 months of zero healthcare‐acquired MRSA infection in all 3 intensive care units (36 beds). We reduced our MRSA‐positive culture from a mean of 30 in 2005–2006 to a mean of 21 in 2007–2008. The Joint Commission has recognised our institution for best practices in infection prevention
4	Nurses’ Use of Positive Deviance When Encountering Electronic Health Records‐Related Unintended Consequences (Bristol et al., 2018)	USA	To study nurses’ experiences with the unintended consequences of using an Electronic Health Record (EHR)	*N* = 144 nurses working for various healthcare organisations	Qualitative descriptive methods. Survey with quantitative questions and 5 open‐ended qualitative questions	X	X	X	Nurses’ experiences with EHR systems offer insight into an organisation's shift toward Resilience Engineering (RE). The ability to recognise the unique needs of nurses during design and implementation of an EHR system may support better resilience in nurses. EHR enhancements based on the results of this research could facilitate better patient care through improved nursing use of the EHR and improved patient safety applications
5	Positive deviance and hand hygiene of nurses in a Quebec hospital: What can we learn from the best? (Létourneau et al., 2018)	Canada	To study PD at the level of a care team, to shed light on dynamics within the group	*N* = 21 nurses (*N* = 6 medical‐surgery unit) (*N* = 15 palliative care unit) at a Montreal university hospital	Focused ethnography design. Systematic observations, individual interviews, field notes, and informal conversations	X		X	It can be useful to apply the positive deviance approach to healthcare teams rather than individuals to better understand the ideologic and structural differences linked to better hand‐hygiene performance by nurses
6	How is success achieved by individuals innovating for patient safety and quality in the NHS? (Sheard et al., 2017)	UK	To study how individuals working in the NHS manage to implement innovations that benefit patient safety	*N* = 15 Health Services Journal (HSJ) innovators (selected from the awards list of 2014 and 2013 working in the area of patient safety and quality in the NHS)	Exploratory qualitative research design Semi‐structured in‐depth interviews	X	X	X	Main factors: i) personal determination of individuals, including their ability to challenge the status quo, ii) their capacity to connect people and teams and encourage collaborative working, iii) the ways in which some innovators used organisational culture to their advantage and iv) using evidence to influence others. While innovation in health care seems hard to achieve, we have uncovered several key aspects which we believe may lead to successful innovation by individuals working in the NHS
7	Positive deviance: a program for sustained improvement in hand‐hygiene compliance (Marra et al., 2011)	Brazil	To study the sustainability of a PD strategy for improving hand‐hygiene compliance in two similar adult stepdown units (SDUs) using electronic handwashing counters	All healthcare workers of two 20‐bed adult SDUs with the same physical layout	Observational study	X	X	X	Based on our findings, PD can be considered an intervention to sustain improved hand‐hygiene compliance and can be associated with a decreased incidence of device‐associated hospital acquired infections
8	Improving the safety and quality of nursing care through standardized operating procedures in Bosnia and Herzegovina (Ausserhofer et al., 2016)	Bosnia and Herzego‐vina	To study if a consistent approach/model was used for development, adaptation, implementation, monitoring and evaluation of nursing standard operating procedures (SOPs)	*N* = 4 healthcare facilities: *N* = 1 hospital and *N* = 1 primary healthcare centre in Republic of Srpska, and *N* = 1 hospital and *N* = 1 primary healthcare centre in Fed. of Bosnia and Herzegovina	Multiple‐case study design, that is an in‐depth empirical inquiry	X		X	The certification/accreditation process is enabling necessary changes in institutions’ organisational cultures, empowering nurses to take on advanced roles in improving the safety and quality of nursing care
9	Positive deviance: Using a nurse call system to evaluate hand‐hygiene practices (de MacEdo et al., 2012)	Brazil	To study the application of PD in 2 stepdown units (SDUs) and evaluate the adherence of nursing staff to hand‐hygiene practices based on the ratio between the number of uses of alcohol‐based hand rub and the number of nurse visits to patient rooms	*N* = 2 SDUs in Albert Einstein Hospital in São Paulo, Brazil. East SDU is a 22‐bed unit for patients with mixed clinical conditions; west SDU is a 22‐bed unit for patients with cardiovascular conditions	Quasi‐experimental study	X		X	The PD approach to hand hygiene produced positive results in terms of compliance to this practice, with increased consumption of alcohol hand rubs, improved ratio of alcohol rub use to nurse visits to patient rooms in the east SDU, and a>2 ratio in both the east and west SDUs. Using this approach led to a reduction in the rate of device‐related infections in both units, with sustained results over 2 years
10	Hospital Strategies for Reducing Emergency Department Crowding: A Mixed‐Methods Study (Chang et al., 2018)	USA	To study strategies among high‐performing, low‐performing and high performance‐improving hospitals to reduce ED crowding, using a PD methodology	2619 hospitals that reported both ED length of stay and boarding time metrics to CMS Hospital Compare in 2012 Interviews, *N* = 60 staff members, including hospital executives, ED chairs and directors, nurse managers, and hospitalists	Mixed‐methods comparative case study			X	Organisational characteristics are associated with ED decreased length of stay. Specific interventions targeted to reduce ED crowding were more likely to be successfully executed at hospitals with these characteristics. These organisational domains represent identifiable and actionable changes that other hospitals may incorporate to build awareness of ED crowding
11	Creating a culture of innovation in nursing education through shared vision, leadership, interdisciplinary partnerships, and positive deviance (Melnyk & Davidson, 2009)	USA	To study barriers and facilitators to innovation in colleges of nursing and healthcare professions along with recommendations for creating a culture of innovation in these academic settings	N.A.	N.A.	X		X	A shared vision for innovation by faculty and staff in colleges of nursing and health sciences is essential to drive innovative cultures, programmes and initiatives. Aligning the vision to measurable goals and outcomes, role modelling innovation, facilitating interdisciplinary collaboration and encouraging positive deviance and risk taking are key ingredients for success. Cultures take time to change. Patience and persistence in working through ‘character‐building’ times are needed to achieve the outcomes established as part of the vision
12	Hospital nurse administrators in Japan: a feminist dimensional analysis (Brandi & Naito, 2006)	Japan	To study key findings from a qualitative study that explored the views of 16 Japanese senior nurse administrators in hospitals to learn what was happening in their working situations and how they were managing	*N* = 16 female participants, including *N* = 1 nursing vice president, *N* = 14 nursing directors and *N* = 1 assistant director, from middle or large‐sized hospitals. Hospital types: private (*N* = 11), public (*N* = 5), general (*N* = 14), specialty (*N* = 2) and university (*N* = 4)	Dimensional analysis strategies for data collection and analysis. Semi‐structured interviews	X		X	Nursing administration as a recognised specialty must rapidly develop to bring nursing and midwifery to the forefront of international healthcare delivery. Nurse administrators are in a position to challenge tradition, but they need advanced education, mentorship and the support of their organisations to enact a role that meets today's goals of patient‐centred care
13	A qualitative positive deviance study to explore exceptionally safe care on medical wards for older people (Baxter et al., 2019)	UK	To study how multidisciplinary teams deliver exceptionally safe care on medical wards for older people (i.e. perform best on a broad safety outcome)	*N* = 70 multidisciplinary staff from 8 medical wards for older people clustered in 13 NHS Trusts in the Yorkshire and the Humber region of England	Qualitative PD study. Focus groups and brief field notes	X	X	X	There are no ‘silver bullets’ to achieving exceptionally safe patient care on medical wards for older people. Healthcare leaders should encourage truly integrated multidisciplinary ward teams where staff know each other and work well together. Focusing on underpinning characteristics may facilitate exceptional performances across a range of safety outcomes
14	Reducing Infections ‘Together’: A review of Socioadaptive Approaches (Sreeramoju, 2019)	N.A.	To study modern‐day physicians and physicians in training expected to participate in interventions to reduce hospital acquired infections and for those who serve as physician champions or lead these initiatives, to gain an understanding of socioadaptive approaches that help reduce these infections	N.A.	N.A.	X		X	Socioadaptive interventions are necessary additions to technical interventions in an overall multicomponent strategy to reduce healthcare‐associated infections. Assessment of local social and cultural context and needs is key to choosing the right socioadaptive approach for any improvement initiative
15	People, systems and safety: resilience and excellence in healthcare practice (Smith & Plunkett, 2019)	N.A.	To study the evolution of safety science, describing historical approaches, comparing them with recent concepts in safety, and describing how they affect staff working in the healthcare system	N.A.	N.A.	X		X	The unspoken expectation is that healthcare practitioners should undertake three roles: 1) to take on the clinical function for which they are engaged, whatever that might be; 2) to not only maintain and enhance patient safety in their own work but also by intervening when needed in their organisational systems; and 3) to seek out opportunities for improving quality and make sure that positive changes are made
16	Using a Positive Deviance Approach to Influence the Culture of Patient Safety Related to Infection Prevention (Sreeramoju et al., 2018)	USA	To study the impact of PD on the patient safety culture related to infection prevention among healthcare personnel	*N* = 6 wards in Parkland Memorial Hospital, an academic medical centre in Dallas. All nurses, patient care technicians, ward managers, and clerks and all patients receiving care in the study wards were included	Observational prospective study with a retrospective baseline period. Outcome of PD intervention was measured with the hospital survey of patient safety climate, adapted to infection prevention	X		X	A positive deviance approach appeared to have a significant impact on patient safety culture among healthcare personnel who received the intervention. Social network analysis identified healthcare personnel who are likely to help disseminate infection prevention information. System‐wide interventions independent of PD resulted in hospital acquired infections reduction in both intervention and control wards
17	Identifying positively deviant elderly medical wards using routinely collected NHS Safety Thermometer data: an observational study (Baxter et al., 2018)	UK	To study a pragmatic method for identifying positively deviant wards using a routinely collected, broad measure of patient safety	Phase 1: *N* = 34 elderly medical wards clustered in *N* = 13 NHS Trusts in the northern region of England, UK Phase 2: Multidisciplinary staff (*N* = 161) and patients (*N* = 188) clustered in *N* = 9 positively deviant and comparison wards	Two‐phased observational study. Phase 1, cross‐sectional and temporal analyses of Safety Thermometer data. Phase 2, multidisciplinary staff and patient surveys	X	X	X	A distinct group of positively deviant wards that perform exceptionally well on a routinely collected, broad measure of safety can be identified using a robust yet pragmatic method. Staff and patient perceptions of safety mainly support their identification. The study highlights the challenges faced when selecting a source of routinely collected data that provides a valid and reliable measure at the appropriate level in order to facilitate performance comparisons across wards or units in several organisations
18	Positive Deviance: A New Tool for Infection Prevention and Patient Safety (Marra et al., 2013)	N.A	N.A.	N.A.	N.A.	X	X	X	The PD approach is particularly appropriate in situations where organisations can track the results with valid performance measures and where there is substantial natural variation in performance. This creates a good environment for discussion of practices and interventions to achieve improvements in patient safety
19	Exploring the concept and use of positive deviance in nursing (Gary, 2013)	N.A.	To study the essence of PD in the nursing practice environment, using the Walker and Avant procedure for concept analysis	N.A.	Concept analysis of positive deviance	X	X	X	The goal was to provide an operational definition for the concept of positive deviance in nursing practice, which can offer nurses a basis for decision‐making when the normal or expected actions in a given situation collide with the nurse's view of the right thing to do. As nurses become more autonomous providers of primary healthcare services, the use of positive deviance must become a goal
20	Positive deviance: An elegant solution to a complex problem (Lindberg & Clancy, 2010)	USA	To study one example of how concepts taken from complex systems theory can be applied to real‐world problems facing nurses today	N.A.	N.A.	X	X		
21	Diamonds in the rough: positive deviance and complexity (Clancy, 2010)	N.A.	To study the idea of PD and how it can be applied in developing elegant solutions to complex problems	N.A.	N.A.	X	X		
22	Positive deviance: a different approach to achieving patient safety (Lawton et al., 2014)	N.A.	N.A.	N.A.	N.A.	X	X		A myopic focus on errors, harm and near misses has long been sending negative messages. Politicians, bureaucrats, managers, the media and those leading enquiries as far back as Bristol Royal Infirmary and earlier, and more recently Mid‐Staffordshire, have essentially indicated to clinicians: you are prone to making mistakes, and we must insist that you reduce the harm or potential harm you cause. If you do not, we will regulate your activities, tightening the rules over time. While no one would argue against the need to identify those people and organisations whose performance is consistently or deliberately negatively deviant, there is a clear obligation to recognise that health care is delivered in complex, uncertain settings, and although clinicians are time‐pressured and resource‐constrained, things go right very often, even in times of austerity
23	Positive deviance: innovation from the inside out (Jaramillo et al., 2008)	N.A.	To study PD theory and how it relates to innovation; an ever‐present need for transformational leaders	N.A.	N.A.		X	X	Positive deviance is a powerful strategy for nursing leaders to effect positive change. This is especially relevant for those on the Magnet journey. We present 7 strategies to assist leaders in recognizing positive deviants in the current environment and for optimizing innovation provide guidance for both experienced and emerging leaders. These strategies support a culture in which creativity, collaboration and knowledge sharing are essential for optimal performance
24	Walking the tightrope: how rebels ‘do’ quality of care in healthcare organisations (Wallenburg et al., 2019)	NL	To study how healthcare professionals and managers give shape to the increasing call for compassionate care as an alternative for system‐based quality management systems	3 Dutch hospitals, studying clinical groups identified as deviant: a ward for infectious diseases, a mother–child department and a dialysis department	Ethnographic research 120 h of observation, 41 semi‐structured interviews and 2 focus groups.	X	X	X	Rebels’ quality practices are an emerging set of collaborative activities to improve health care and meet (individual) patient needs. Rebels conduct ‘contexting work’ to achieve their quality aims by expanding their normative work to outside domains. As rebels deviate from hospital policies, they are sometimes forced to act ‘under the radar’, risking ‘groupthink’ and may undermine the aim of public accounting
25	Nurse managers: Being deviant to make a difference (Crewe & Girardi, 2020)	Australia	To study how positive nurse‐manager behaviours that deviate from ‘business as usual’ promote positive nursing outcomes	*N* = 7 nurse managers from a private hospital in Australia and *N* = 17 from the public health sector in Seychelles	An interpretivist methodology	X	X	X	Study addresses the call for the ‘study of positive outcomes, processes, and attributes of organizations and their members’ deemed valuable in health care. Interview data support that positive leadership strategies and practices that facilitate meaningful work, relationships, positive climates and supportive communication, can impact organisational and individual outcomes. Importantly, positive leadership, not just interventions alone, leads to interventions that influence organisational outcomes

N.A., not available.

### 3.3 Quality appraisal

To evaluate the quality of the included papers and the degree of evidence in a transparent and unbiased way, the research methodology (see Appendix S2) involved using the Mixed Methods Appraisal Tool (MMAT; Hong et al., 2018). However, quality as such was not a criterion to exclude papers from the review. The quality appraisal was conducted independently by two researchers (EdK and AW).

## 4 RESULTS

**FIGURE 1:**
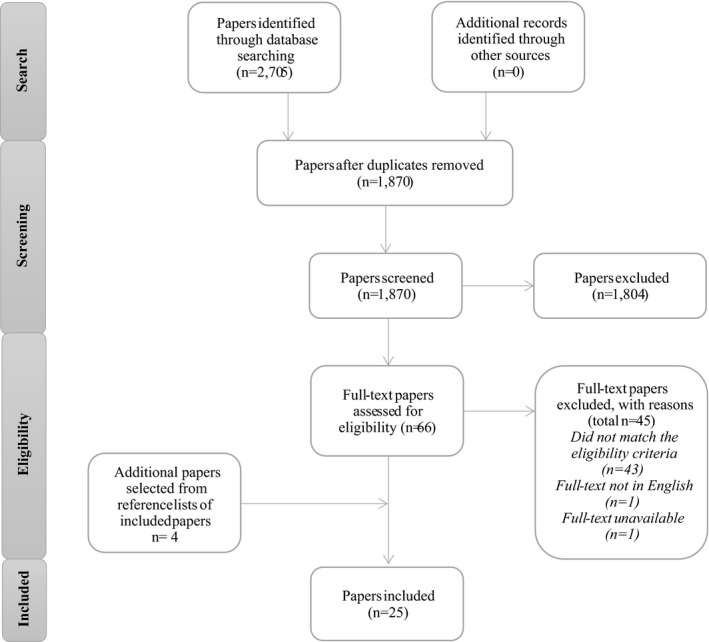
Flow chart of inclusion process

The initial search strategy generated 2705 papers (Figure 1). After removing duplicates and screening the titles and abstracts in the first stage of screening, 66 papers were selected. In the second stage, all 66 papers were read in full, and following assessment, 21 papers were agreed upon for inclusion. The references of these 21 papers were reviewed, and four relevant papers were added. In total, 25 papers were analysed further. Table 1 presents the data from these papers. Because of the wide variety of methodological approaches, we present the content findings of our scoping review as narratives. Below we discuss the three concepts (positive deviance, healthcare rebels and tempered radicals) separately and show their similarities.

### 4.1 Descriptions of the concepts

In the 25 selected papers, ‘positive deviance’ was mentioned 23 times and ‘tempered radicals’ (Brandi & Naito, 2006) and ‘healthcare rebel’ (Wallenburg et al., 2019) once each. Content analysis of the various descriptions showed that three aspects are often mentioned (Table 2).

**TABLE 2:** Results: descriptions, competences and stimulating/hindering factors

	Paper	Concept	Descriptions			Competences			Stimulating/hindering factors			
			PD approach	Behaviour	Networks/Relationships	Collaborate	Expert (evidence‐based) knowledge	Courage/Challenge status quo	Dialogues/Reflection	Networking	Role of management	Hindering factors
1	Combating infections at Maine Medical Center: Insights into complexity‐informed leadership from positive deviance (Lindberg & Schneider, 2013)	PD	X	X	X	X			X	X	X	
2	Beyond the hospital infection control guidelines: A qualitative study using positive deviance to characterise grey areas and to achieve efficacy and clarity in the prevention of healthcare‐associated infections (Gesser‐Edelsburg et al., 2018)	PD	X	X	X				X			
3	Methicillin‐resistant Staphylococcus aureus (MRSA) prevention through facility‐wide culture change (Bonuel et al., 2009)	PD	X	X	X						X	
4	Nurses’ Use of Positive Deviance When Encountering Electronic Health Records‐Related Unintended Consequences (Bristol et al., 2018)	PD	X	X	X	X	X	X				X
5	Positive deviance and hand hygiene of nurses in a Quebec hospital: What can we learn from the best? (Létourneau et al., 2018)	PD	X	X	X				X			
6	How is success achieved by individuals innovating for patient safety and quality in the NHS? (Sheard et al., 2017)	PD	X	X	X	X	X	X				X
7	Positive deviance: a programme for sustained improvement in hand‐hygiene compliance (Marra et al., 2011)	PD	X		X	X	X		X			
8	Improving the safety and quality of nursing care through standardised operating procedures in Bosnia and Herzegovina (Ausserhofer et al., 2016)	PD	X								X	
9	Positive deviance: Using a nurse call system to evaluate hand‐hygiene practices (de MacEdo et al., 2012)	PD	X						X			
10	Hospital Strategies for Reducing Emergency Department Crowding: A Mixed‐Methods Study (Chang et al., 2018)	PD									X	
11	Creating a culture of innovation in nursing education through shared vision, leadership, interdisciplinary partnerships, and positive deviance (Melnyk & Davidson, 2009)	PD		X					X			
12	Hospital nurse administrators in Japan: a feminist dimensional analysis (Brandi & Naito, 2006)	Tempered radicals		X						X	X	X
13	A qualitative positive deviance study to explore exceptionally safe care on medical wards for older people (Baxter et al., 2019)	PD	X						X	X		
14	Reducing Infections ‘Together’: A review of Socioadaptive Approaches (Sreeramoju, 2019)	PD	X		X				X			
15	People, systems and safety: resilience and excellence in healthcare practice (Smith & Plunkett, 2019)	PD		X					X			
16	Using a Positive Deviance Approach to Influence the Culture of Patient Safety Related to Infection Prevention (Sreeramoju et al., 2018)	PD	X		X				X		X	
17	Identifying positively deviant elderly medical wards using routinely collected NHS Safety Thermometer data: an observational study (Baxter et al., 2018)	PD	X			X	X		X			
18	Positive Deviance: A New Tool for Infection Prevention and Patient Safety (Marra et al., 2013)	PD	X		X	X	X	X	X	X		
19	Exploring the concept and use of positive deviance in nursing (Gary, 2013)	PD	X	X	X	X	X	X				
20	Positive deviance: An elegant solution to a complex problem (Lindberg & Clancy, 2010)	PD	X		X	X						
21	Diamonds in the rough: positive deviance and complexity (Clancy, 2010)	PD	X	X	X	X	X	X				
22	Positive deviance: a different approach to achieving patient safety (Lawton et al., 2014)	PD			X	X						
23	Positive deviance: innovation from the inside out (Jaramillo et al., 2008)	PD						X			X	
24	Walking the tightrope: how rebels ‘do’ quality of care in healthcare organisations (Wallenburg et al., 2019)	Healthcare rebels		X	X	X	X	X	X	X	X	X
25	Nurse managers: Being deviant to make a difference (Crewe & Girardi, 2020)	PD		X	X	X			X			

Abbreviation: PD, positive deviance.

Most of the studies identify positive deviant healthcare professionals, departments and/or organisations. Determining who the positive deviants are is done by researchers (Gesser‐Edelsburg et al., 2018; Sheard et al., 2017), by colleagues (Gesser‐Edelsburg et al., 2018; Lawton et al., 2014; Marra et al., 2011) and performance figures; for example, hospitals that are within the top and bottom 5% of Centers for Medicare and Medicaid services (Baxter et al., 2018, 2019; Chang et al., 2018; Létourneau et al., 2018). In the healthcare rebel study, performance figures and public opinion were used to select the healthcare organisation while colleagues selected the rebel groups (Wallenburg et al., 2019). However, the methodology used to assess or determine positive deviants, healthcare rebels and tempered radicals by researchers and colleagues is seldom described. Only the study by Wallenburg et al. (2019) mentioned interviews with colleagues. Despite the unclear methodology, most papers define positive deviants, healthcare rebels, tempered radicals and their competences.

#### 4.1.1 Concept descriptions

Most of the papers (17/25) describe using positive deviance as a method to initiate conscious and systematic (behavioural) change in an organisation. The positive deviance method is based on the assumption that in each community, individuals or groups can find better solutions and achieve better results than their peers by executing unusual behaviour even though the circumstances and availability of materials and resources are the same for all (Ausserhofer et al., 2016; Baxter et al., 2018; Bonuel et al., 2009; Bristol et al., 2018; Clancy, 2010; de MacEdo et al., 2012; Gary, 2013; Gesser‐Edelsburg et al., 2018; Létourneau et al., 2018; Lindberg & Clancy, 2010; Lindberg & Schneider, 2013; Marra et al., 2011, 2013; Sheard et al., 2017; Sreeramoju, 2019; Sreeramoju et al., 2018). These individuals or groups can be identified and pushed forward, and organisations can learn from their approaches (Baxter et al., 2018, 2019). Wallenburg et al. (2019) use the term rebels to describe the same deviant behaviour in healthcare professionals striving for the best quality. Rebels (groups) consciously deviate to accomplish change in organisations. Brandi and Naito (2006) complement this view by noting that tempered radicals pursue changes that go against the norms of dominant groups for better results.

To achieve better results under the same circumstances, positive deviants demonstrate behaviour and working methods that deviate from the norm (Clancy, 2010; Crewe & Girardi, 2020; Gary, 2013; Létourneau et al., 2018; Lindberg & Schneider, 2013; Melnyk & Davidson, 2009; Smith & Plunkett, 2019). The study on tempered radicals also mentions this (Brandi & Naito, 2006). The behaviour of deviating healthcare professionals is often described in the literature as unconventional and non‐confirmative behaviour (Bonuel et al., 2009; Gary, 2013; Gesser‐Edelsburg et al., 2018; Lindberg & Schneider, 2013; Melnyk & Davidson, 2009; Sheard et al., 2017; Wallenburg et al., 2019). The study by Bristol et al. (2018) shows, for example, that nurses display positive abnormal behaviour when faced by system requirements of an electronic patient record that do not meet the needs of the patient. Nurses make various ‘workarounds’ to meet their patient's needs and do not comply with the restrictions of the electronic patient record (Bristol et al., 2018).

The literature on both positive deviants and healthcare rebels describes the relevance of social networks and personal relationships in and outside the organisation (Bristol et al., 2018; Crewe & Girardi, 2020; Gary, 2013; Gesser‐Edelsburg et al., 2018; Lawton et al., 2014; Létourneau et al., 2018; Lindberg & Clancy, 2010; Lindberg & Schneider, 2013; Sheard et al., 2017; Wallenburg et al., 2019). These networks and relationships spread successful practices, allowing nurses to share strategies and ideas (Bonuel et al., 2009; Gary, 2013; Létourneau et al., 2018; Lindberg & Clancy, 2010; Marra et al., 2013). Positive deviants and healthcare rebels often serve as influential role models who can exert peer pressure in these networks (Clancy, 2010; Gary, 2013; Marra et al., 2011; Sreeramoju, 2019; Sreeramoju et al., 2018).

#### 4.1.2 Competences

Of the 25 papers, 15 describe the competences of healthcare rebels, positive deviants or tempered radicals (Table 1). The most frequently mentioned competence is the ability to collaborate and network (12/25 papers; Table 2; Baxter et al., 2018; Bristol et al., 2018; Clancy, 2010; Crewe & Girardi, 2020; Gary, 2013; Lawton et al., 2014; Lindberg & Clancy, 2010; Lindberg & Schneider, 2013; Marra et al., 2011, 2013; Sheard et al., 2017; Wallenburg et al., 2019). Deviating healthcare professionals collaborate with peers (i.e. nurse colleagues in the same position), colleagues from diverse disciplines or in management positions (Crewe & Girardi, 2020; Lindberg & Clancy, 2010; Sheard et al., 2017; Wallenburg et al., 2019) and colleagues from other departments and even other organisations (Bristol et al., 2018; Clancy, 2010; Lindberg & Schneider, 2013; Wallenburg et al., 2019). Deviating healthcare professionals know who to approach in their large network when help is needed (Wallenburg et al., 2019). Also mentioned are the competences to connect people and encourage others to take ownership of a problem (Clancy, 2010; Gary, 2013; Lawton et al., 2014; Marra et al., 2011; Sheard et al., 2017).

Other competences include using expert knowledge, scientific evidence, to improve care. Healthcare professionals who deviate actively seek evidence and spread this information. Therefore, colleagues regard them as experts and valuable, reliable sources of information (Bristol et al., 2018; Clancy, 2010; Gary, 2013; Marra et al., 2013; Wallenburg et al., 2019). If positive deviants want to convince others, they use collected data or scientific evidence (Baxter et al., 2018; Marra et al., 2011; Sheard et al., 2017).

Healthcare rebels characteristically have the courage to challenge the status quo (Wallenburg et al., 2019). Marra et al. (2013) describe this as an ability to reflect on working habits, organisational logistics and problems in daily care and generate ideas to improve care. Deviating healthcare professionals are determined to improve (Gary, 2013; Sheard et al., 2017) and dare to stretch the boundaries by for example breaking the rules (Gary, 2013). Wallenburg et al. (2019) describe how they make trade‐offs between short‐term improvements by breaking the rules and disobeying regulations while trying to achieve a more structural solution so that deviance is no longer needed.

The solutions to complex problems are often sold as elegant and efficient (Bristol et al., 2018). According to Gary (2013) and Wallenburg et al. (2019), deviating from the norm or breaking the rules is always done in the interests of the patient and the aim is to find better ways to get things done with the same or fewer resources (Jaramillo et al., 2008; Marra et al., 2013). Despite their deviant behaviour, rebels are committed to the mission and goals of the organisation and want to provide the best care (Gary, 2013).

Research shows nurses do not always see themselves as a positive deviant, healthcare rebel and or tempered radical (Lindberg & Schneider, 2013). Sometimes, in talking about their work and what they do, they discover that they are deviant or rebellious. Thus, this kind of leadership is often unconscious and unintentional (Lindberg & Schneider, 2013).

In summary, based on the descriptions and competences described above, rebel nurse leaders can be characterised as networkers who collaborate with their peers, other disciplines and management in and outside the organisation, using both formal and informal conversations. They are seen as experts based on their (evidence‐based) knowledge. Their courage and competence in reflection help them to challenge the current status quo, deviating from the rules and regulations to achieve their goal of (solving problems which) improve daily care in both the short and longer term.

### 4.2 Factors stimulating and hindering the development of rebel nurse leadership

The included papers were also screened for factors that stimulate or hinder the development of rebel nurse leadership. 22 of the 25 papers describe three important factors (Table 2).

#### 4.2.1 Dialogue and reflection

In the positive deviance literature, deviance is stimulated by organising and conducting planned conversations such as meetings (Crewe & Girardi, 2020; de MacEdo et al., 2012; Létourneau et al., 2018; Lindberg & Schneider, 2013; Marra et al., 2011, 2013; Sreeramoju et al., 2018), structured reflective dialogue, and informal and spontaneous conversations (Sreeramoju, 2019). An example mentioned in the literature of a planned conversation is a Discovery and Action Dialogues (DAD; Lindberg & Schneider, 2013). DAD are small‐grouped facilitated conversations with healthcare professionals from different professional backgrounds to identify positive deviant practices on a specific topic. The aim of these DAD is to reveal positive deviance actions and to discuss the obstacles for broader implementation.

Sharing experiences with professionals from different backgrounds support personal relationships and understanding and respect for one another, resulting in improved collaboration (Baxter et al., 2018; Lindberg & Schneider, 2013). Wallenburg et al. (2019) found that in planned conversations, healthcare rebels reveal their normativity and the normative work involved in what they consider is ‘good’ care and how it should be organised. Papers mention that professionals feel heard in conversations, with an openness encourages them to talk about the problems they encounter and share new insights and solutions to improve the quality of care (Lindberg & Schneider, 2013; Melnyk & Davidson, 2009). Research by Sreeramoju et al. (2018) adds the importance of confidence in both formal reflective dialogue and informal conversations. Smith and Plunkett (2019) explain the relevance of a work environment in which professionals feel safe, so they dare to ask reflective questions, ask for help and take risks. An important effect of spreading new ideas and actions is an environment of eagerness to find even more constructive ideas (Gesser‐Edelsburg et al., 2018).

#### 4.2.2 Networking

In the positive deviance approach, networks are used to spread new ideas and deviant actions. These are the individuals’ own networks and/or developed in conversations, both structured (as DAD) and informal (Brandi & Naito, 2006; Lindberg & Schneider, 2013; Marra et al., 2013). The paper on tempered radicals elaborates on networking and describes collaborations and alliances to change things by finding likeminded people and supportive relationships (Brandi & Naito, 2006).

Baxter et al. (2019, p. 622) describe the nature of these networks: ‘It helped them to support one another to deliver safe patient care. Friendly, personal connections between staff members were perceived to facilitate dialogue, influence their ability to contribute different perspectives, encourage them to work beyond silos and to be more broadly involved in patient care’. In the rebel paper, networking is a part of what they call ‘contexting, [which] is about networking and encouraging others to act in line with rebels’ practices of caring’ (Wallenburg et al., 2019, p. 877).

#### 4.2.3 Role of management

Although the positive deviance approach seems to be a bottom‐up movement, several papers show the importance of management involvement and support (Ausserhofer et al., 2016; Bonuel et al., 2009; Chang et al., 2018). This is also found in the literature on tempered radicals (Brandi & Naito, 2006) and healthcare rebels (Wallenburg et al., 2019). Managers play a role in stimulating dialogue among professionals, by asking critical questions, challenging the current status quo and stimulating rebel behaviour if they feel things can be improved (Wallenburg et al., 2019). Especially in the rebel paper (Wallenburg et al., 2019), managers show the same kind of behaviour as healthcare rebels.

The respect and endorsement of CEOs and support by middle managers prevent healthcare professionals from being penalised by powerful people in the organisation who might view deviant behaviour as intrusive, threatening or inappropriate (Lindberg & Schneider, 2013). Knowing management has your back allows healthcare professionals to talk about their deviant actions to innovate and improve patient care (Jaramillo et al., 2008) without fearing negative consequences (Sreeramoju et al., 2018). Management can also play a pivotal role in promoting deviant action and to spread these good practices (Lindberg & Schneider, 2013).

#### 4.2.4 Hindering factors

A few papers (4/25) describe the obstacles to being a positive deviant, healthcare rebel or tempered radical. First, a nurse who acts like a positive deviant risks facing negative perceptions by management, punishment and the ultimate consequence of losing their job or licence (Bristol et al., 2018). The fear of damage to their reputation or career keeps healthcare professionals from talking about deviating from organisational policy (Lindberg & Schneider, 2013). Wallenburg et al. (2019) observed the same; healthcare rebels ‘stay under the radar’ to avoid these negative consequences. However, keeping deviant actions hidden impedes the innovative spirit and ability to spread the innovation (Sheard et al., 2017; Wallenburg et al., 2019). The research by Brandi and Naito (2006) describes the harmful consequences for the organisation and/or the individual tempered radical nurse who may feel ‘trapped’ in their position. ‘Alternatives to tempered radicalism are to seek other jobs, surrender to silence and disempowerment, or assimilate to the conflicting dominant viewpoint or values of an organization’ (Brandi & Naito, 2006, p. 64).

Put together, the supporting factors are as follows: (1) confidential conversations (both planned and spontaneous) and reflective dialogue in a safe work environment to reveal positive deviant behaviour of rebel leadership support the exchange of normative points of view on the current situation and collectively find new solutions for points of improvement; (2) networking in and outside the organisation to spread the deviant actions and ideas that help to encourage others; and (3) management respect and support that stimulates professionals to deviate. Hindering factors are the negative consequences for personal reputation and/or career that urge professionals deviating from the rules and regulations to ‘stay under the radar’.

## 5 DISCUSSION

This scoping review provides an overview of (1) descriptions of positive deviance, healthcare rebels and tempered radicals in nursing; (2) the competences of rebel nurse leaders; and (3) factors that stimulate the development of rebel nurse leadership.

Most studies included in the review identify positive deviant healthcare professionals, departments and/or organisations. However, they seldom describe their exact selection criteria. Selection is methodologically challenging, as deviant or rebel behaviour requires a comparison with something regarded as ‘normal’ and is thus highly normative depending on the eye of the beholder. Besides, the methodology applied to ‘organise’ learning from positive deviant behaviour and/or methods might not be all‐encompassing as healthcare professionals prefer or even need to ‘stay under the radar’ to perform deviant behaviour (Wallenburg et al., 2019). Additionally, the selected papers make no mention of ‘negative’ deviation as all perspectives are highly appreciative on the topic. Numerous studies showed that deviating from clinical guidelines (without a proper reason) results in low quality of care (e.g. Rice et al., 2012; Sargen & Kingsnorth, 2001).

Nevertheless, the findings of this scoping review demonstrate a variety of descriptions and definitions on positive deviance, healthcare rebels and tempered radicals. Analysing these descriptions and definitions has made the overlap between these concepts apparent. All descriptions in the literature focus on deviant behaviour by healthcare rebels who, as a result, achieve better outcomes under the same circumstances than their peers, according to the authors. Only a few authors specify the better outcomes (by making a comparison), and no paper shows evidence that these better outcomes can be attributed to the positive deviant(s), healthcare rebel(s) or tempered radical(s) studied. Only the study by Wallenburg et al. (2019) used ethnographic methodology (observations, informal conversations and semi‐structured interviews), to study more ‘objectively’ the results of rebel leadership.

Although the concepts of positive deviance, healthcare rebels and tempered radicals are similar, they also have important differences. The positive deviance approach purposefully identifies positive deviants, makes them visible and gives them an exemplary role with the aim of learning from them. Healthcare rebels or tempered radicals, on the other hand, are less visible in organisations as they prefer to ‘stay under the radar’ to avoid criticism (Wallenburg et al., 2019). Allen (2014) describes this as the invisible work of nurses to ‘keep things on track’ and serve as a ‘Jack of all trades’. Our study focuses on rebel nurse leadership from the perspective of ‘good rebels’ who can ‘rock the boat while staying in it’, as Bevan (2010) and Meyerson (2008) put it. The ‘bad rebels’ who deviate and break the rules for personal gains or because of angry assertations and complaints were hardly mentioned in the included literature. There is a thin line between a ‘good’ and ‘bad’ rebel, and assessing the difference is a subjective matter. This matter fell outside the scope of our review; more empirical research in this direction would help enrich the literature on rebel (nurse) leadership.

LAP theory (Raelin, 2011) helps us to understand how leadership is enacted in the nursing workplace and how the context influences leadership and the dynamics within organisations that foster leadership (Raelin, 2011). However, the included papers do not describe the daily practices of nurses in terms of LAP, and thus, it is unclear what is actually enacted in the practices of positive deviants, healthcare rebels and tempered radicals. We regard this lack of transparency on the context and dynamics within the organisations concerned as a missed opportunity.

Further, we expected more papers to describe the practices of healthcare rebels, as the work of Helen Bevan and her colleagues has resonated in the healthcare sector worldwide. Bevan's educational programme, School for Health and Care Radicals, was launched by the UK National Health Service (NHS) in 2014. The purpose of this education programme is to teach employees ‘to rock the boat and stay in it’ (Bevan & Fairman, 2016). The School for Health and Care Rebels extended ‘beyond the NHS and across global healthcare networks. More than 1,500 people enrolled across 40 countries’ in its first year (Nesta, 2014). In the years following it was transformed into the School for Change Agents, offering free webinars and modules in a Massive Online Open Course (Bevan, 2018). Despite all the attention, there is a lack of scientific papers describing the practices of nurse rebel leaders and healthcare organisations that deliberately support the development of these healthcare rebels. This limits our knowledge about the programme. Only Grifford et al. (2015) have studied the results of the programme and changes in work environments of nurses after the first year. Unfortunately, only one paper in our scoping review (Wallenburg et al., 2019) describes the practices of healthcare rebels. As mentioned earlier, one reason for this lack of information might be the difficulty of studying nurse rebel leadership and deviant behaviour because rebels tend to ‘stay under the radar’. To capture actual practice is thus challenging.

Most included papers define the competences of positive deviants, healthcare rebels and tempered radicals. This review demonstrated four aspects of competence in rebel nurse leaders, of which two are interpersonal: (1) collaboration (networking); (2) communications (gain and share expertise knowledge and challenge the current status quo); and two are intrapersonal: (3) the ability to critically assess and reflect (on working habits, organisation logistics, problems in daily care); and (4) come up with innovative ideas. Bevan (2013) and Meyerson (2008) also describe these four competences. Note that the competences listed by the included papers are not unique to rebel nurse leaders; they also arise in concepts of leadership both inside and outside healthcare.

Note that one relevant aspect influencing rebel nurse leaders previously described in literature was not found in the 25 papers. According to Meyerson (2008, p. 5), ‘they [tempered radicals] are treated as outsiders because they represent ideals or agendas that are somehow at odds with the dominant culture’. The included papers regard positive deviants, healthcare rebels and tempered radicals as role models for their peers and not as outsiders. Possibly, the ‘outsider’ was not found in the included paper because of their process‐oriented approach to positive deviance methodology.

This scoping review might help researchers bind together the concepts of positive deviance, healthcare rebels and tempered radicals so that studies of nurse rebel leadership will enter the nursing (leadership) literature. Leadership in individual nurses is required when nurses need to balance between being a ‘good’ employee and ‘deviating’ for the benefit of patient care or the organisation. Nurses must streamline processes aimed at better service provision, intertwining the professional and organisational logics as natural aspects of professional action (Noordegraaf, 2015). To gain an understanding of rebel nurse leaders in their daily practice of doing compassionate and good care, studying rebel nurse leadership would be a useful addition to the current nursing leadership literature, especially when blended with LAP.

### 5.1 Strengths and limitations

This scoping review used precise, transparent methods based on study and reporting guidelines (Peters et al., 2017; Tricco et al., 2018). However, it has three limitations. First, most of the included papers concerned positive deviance, describing a positive deviance methodology and the results it gained, without any focus on individual positive deviants. Our data extraction required close reading to understand the relevant competences of rebel nurse leaders and aspects that support or hinder their positive deviant behaviour. We might have put too much emphasis on this, as it was not the stated aim of most of the positive deviant papers. We found very few papers on the concepts of healthcare rebels and tempered radicals in nursing and this also influenced the validity of our findings.

The second limitation is the method of document selection. The selection criteria were designed to include relevant papers focusing on nurses. Hence, papers describing healthcare professional teams or healthcare professionals in general were excluded. However, nurses could have been members of such teams, and as a result, selection bias may have occurred.

The third limitation is that papers not based on scientific research were excluded, which means we may have missed potentially relevant information. For instance, the work of Helen Bevan on healthcare rebels was not included, because she writes only blogs and white papers (Bevan, 2013; Bevan & Fairman, 2016).

Despite these limitations, this is the first scoping review based on a comprehensive literature search to assess the current state of what we call rebel nurse leadership. Our study provides a lens for studying rebel nurse leadership in that it describes what it entails and the competences that contribute to it.

### 5.2 Future research

The findings of this scoping review can be used in further studies on nurse rebel leadership in daily practice to gain more understanding of its influence on improving the quality of care. Shadowing could help accurately describe the practices of rebel nurse leaders and reveal more about their working context, strategies and behaviour (Lalleman et al., 2017).

Exploring the experiences of nurses seen as rebel leaders could be useful. Interviewing these nurses to study their perception and interpretation of rebel nurse leadership would help refine the description of the concept and apply the findings of this review in daily practice.

The stimulating and hindering factors this review describes could also be useful. For instance, studying interventions that foster communication among nurses—dialogue, reflection and networking competences—as well as interventions that change the role of the management could help us understand how these factors influence rebel nursing leadership.

## 6 CONCLUSION

Nurses’ leadership plays a crucial role in daily practice, especially given the current challenge of retaining nurses and maintaining healthcare quality. This scoping literature review aimed to provide an overview of rebel nurse leadership, culled from the literature on positive deviance, healthcare rebels and tempered radicals. Our review gives insights into nurse rebel leadership, describes the competences of rebel nurse leaders and explains the factors that stimulate or hinder the development of rebel nurse leadership.

After synthesising the descriptions and competences mentioned in the three concepts, we identified several common aspects. Rebel nurse leaders show unconventional nonconformist behaviour that varies or differs from norms, rules, codes of conduct, practices or strategies. They challenge the status quo with their ability to develop and use social networks (peers, other disciplines and management) in‐ and outside their organisation to obtain evidence‐based knowledge. They share information and gain the engagement of others to provide better outcomes for patients and organisations. As a result, these nurse leaders consistently outperform their peers using the same resources.

Important competences are the ability to: (1) collaborate and network with diverse professionals and management in‐ and outside the organisation, (2) obtain and share expert (evidence‐based) knowledge, (3) critically reflect on working habits, organisational logistics and problems in daily care and dare to challenge the status quo and (4) generate ideas to improve care. Factors supporting rebel nurse leadership are as follows: (1) formal and informal communication—dialogues and reflection—to reveal positive deviant behaviour, to support the exchange of normative points of view on the current situation and collectively find new solutions to improve quality, (2) networking in‐ and outside the organisation to share deviant activity and ideas that help to encourage others and (3) management's willingness to stimulate professional deviation.

## 7 RELEVANCE TO CLINICAL PRACTICE

This scoping review describes rebel nurse leaders, their competences, and provides an overview of factors that stimulate or hinder the development of rebel nurse leadership. This understanding will help management and nurses to support and develop rebel nurse leadership. More nurse leadership will influence and enhance the quality of care and help retain nurses.

## CONFLICT OF INTEREST

The authors declare that they have no conflicts of interest.

## AUTHOR'S CONTRIBUTION

EdK, PL and AW designed the study. EdK performed the scoping review. EdK, PL and AW analysed and interpreted the data. EdK and AW prepared the manuscript. PL and LS commented on the manuscript. All authors approved the final version for submission.

## Supporting information

Supplementary Material

Appendix S1

Appendix S2
