# Supplementary material for: A scoping review of rebel nurse leadership: Descriptions, competences and stimulating/hindering factors
Source: J Clin Nurs. 2021 May 6;30(17-18):2563–83. doi: 10.1111/jocn.15765 (PMC8453833; doi:10.1111/jocn.15765)
Supplement: Supplementary file 2 — Appendix S1 [file JOCN-30-2563-s002.docx]

Appendix 1. Search strings

*Pubmed*

(((Rebel*) OR Tempered Radical*) OR Positive deviance) AND Health* AND (("1995/01/01"[PDat] : "2020/03/31"[PDat]))

*CINAHL*

Rebel* OR Tempered radical* OR Positive deviance AND Health* Limiters - Published Date: 19950101-20200331 Search modes - Boolean/Phrase

*Scopus*

( TITLE-ABS-KEY ( rebel* )  OR  TITLE-ABS-KEY ( tempered  AND radical* )  OR  TITLE-ABS-KEY ( positive  AND deviance )  AND  TITLE-ABS-KEY ( health* ) )  AND  ( EXCLUDE ( PUBYEAR ,  1994 )  OR  EXCLUDE ( PUBYEAR ,  1993 )  OR  EXCLUDE ( PUBYEAR ,  1992 )  OR  EXCLUDE ( PUBYEAR ,  1991 )  OR  EXCLUDE ( PUBYEAR ,  1990 )  OR  EXCLUDE ( PUBYEAR ,  1989 )  OR  EXCLUDE ( PUBYEAR ,  1988 )  OR  EXCLUDE ( PUBYEAR ,  1987 )  OR  EXCLUDE ( PUBYEAR ,  1986 )  OR  EXCLUDE ( PUBYEAR ,  1985 )  OR  EXCLUDE ( PUBYEAR ,  1984 )  OR  EXCLUDE ( PUBYEAR ,  1983 )  OR  EXCLUDE ( PUBYEAR ,  1982 )  OR  EXCLUDE ( PUBYEAR ,  1981 )  OR  EXCLUDE ( PUBYEAR ,  1980 )  OR  EXCLUDE ( PUBYEAR ,  1979 )  OR  EXCLUDE ( PUBYEAR ,  1978 )  OR  EXCLUDE ( PUBYEAR ,  1977 )  OR  EXCLUDE ( PUBYEAR ,  1976 )  OR  EXCLUDE ( PUBYEAR ,  1975 )  OR  EXCLUDE ( PUBYEAR ,  1974 )  OR  EXCLUDE ( PUBYEAR ,  1973 )  OR  EXCLUDE ( PUBYEAR ,  1972 )  OR  EXCLUDE ( PUBYEAR ,  1971 )  OR  EXCLUDE ( PUBYEAR ,  1970 )  OR  EXCLUDE ( PUBYEAR ,  1969 )  OR  EXCLUDE ( PUBYEAR ,  1968 )  OR  EXCLUDE ( PUBYEAR ,  1966 )  OR  EXCLUDE ( PUBYEAR ,  1965 )  OR  EXCLUDE ( PUBYEAR ,  1964 )  OR  EXCLUDE ( PUBYEAR ,  1961 )  OR  EXCLUDE ( PUBYEAR ,  1955 )  OR  EXCLUDE ( PUBYEAR ,  1954 )  OR  EXCLUDE ( PUBYEAR ,  1933 ) )

*PsycInfo*

((Rebel* or Tempered radical* or Positive deviance) and Health*).mp. [mp=title, abstract, heading word, table of contents, key concepts, original title, tests & measures] limit 3 to yr="1995 -Current"
