# Supplementary material for: A scoping review of rebel nurse leadership: Descriptions, competences and stimulating/hindering factors
Source: J Clin Nurs. 2021 May 6;30(17-18):2563–83. doi: 10.1111/jocn.15765 (PMC8453833; doi:10.1111/jocn.15765)
Supplement: Supplementary file 3 — Appendix S2 [file JOCN-30-2563-s003.docx]

Appendix 2. Quality Appraisal

| **Quality Appraisal of Qualitative studies** | **S1. Are there clear research questions?** | **S2. Do the collected data allow to address the research questions?** | **1.Is the qualitative approach appropriate to answer the research question?** | **2.Are the qualitative data collection methods adequate to address the research question?** | **3.Are the findings adequately derived from the data?** | **4. Is the interpretation of results sufficiently substantiated by data?** | **5. Is there coherence between qualitative data sources, collection, analysis and interpretation?** |
| --- | --- | --- | --- | --- | --- | --- | --- |
| Beyond the hospital infection control guidelines: A qualitative study using positive deviance to characterize gray areas and to achieve efficacy and clarity in the prevention of healthcare-associated infections. (Gesser-Edelsburg et al., 2018) | Yes | Yes | Yes | Yes | No | Yes | Yes |
| Nurses’ Use of Positive Deviance When Encountering Electronic Health Records-Related Unintended Consequences. (Bristol et al., 2018) | Yes | Yes | Yes | Yes | Yes | Yes | Yes |
| Positive deviance and hand hygiene of nurses in a Quebec hospital: What can we learn from the best? (Létourneau et al., 2018) | Yes | Yes | Yes | Yes | Yes | Yes | Yes |
| How is success achieved by individuals innovating for patient safety and quality in the NHS? (Sheard et al., 2017) | Yes | Yes | Yes | Yes | Yes | Yes | Yes |
| Positive deviance: a program for sustained improvement in hand-hygiene compliance. (Marra et al., 2011) | Yes | No | No | No | No | No | No |
| Improving the safety and quality of nursing care through standardized operating procedures in Bosnia and Herzegovina. (Ausserhofer et al., 2016) | Yes | Yes | Yes | Yes | Yes | No | Yes |
| Hospital nurse administrators in Japan: a feminist dimensional analysis. (Brandi & Naito, 2006) | Yes | Yes | Yes | Yes | Yes | Yes | Yes |
| A qualitative positive deviance study to explore exceptionally safe care on medical wards for older people. (Baxter et al., 2019) | Yes | Yes | Yes | Yes | Yes | Yes | Yes |
| Using a Positive Deviance Approach to Influence the Culture of Patient Safety Related to Infection Prevention. (Sreeramoju et al., 2018) | Yes | Yes | Yes | Yes | Yes | Yes | Yes |
| Walking the tightrope: how rebels “do” quality of care in healthcare organizations. (Wallenburg et al., 2019) | Yes | Yes | Yes | Yes | Yes | Yes | Yes |
| Nurse managers: Being deviant to make a difference. (Crewe & Girardi, 2020) | Yes | Yes | Yes | Yes | Yes | Yes | Yes |
| **Quality Appraisal of Non-Randomized studies** | **S1. Are there clear research questions?** | **S2. Do the collected data allow to address the research questions?** | **1. Are the participants representative of the target population?** | **2. Are measurements appropriate regarding both the outcome and intervention (or exposure)?** | **3. Are there complete outcome data?** | **4. Are the confounders accounted for in the design and analysis?** | **5. During the study period, is the intervention administered (or exposure occurred) as intended?** |
| Positive deviance: Using a nurse call system to evaluate hand-hygiene practices. (de MacEdo et al., 2012) | Yes | Yes | Yes | Yes | No | Yes | Yes |
| Identifying positively deviant elderly medical wards using routinely collected NHS Safety Thermometer data: an observational study. (Baxter et al., 2018) | Yes | Yes | Yes | Yes | Yes | Yes | Yes |
| **Quality Appraisal of Mixed methods studies** | **S1. Are there clear research questions?** | **S2. Do the collected data allow to address the research questions?** | **1. Is there an adequate rationale for using a mixed methods design to address the research question?** | **2. Are the different components of the study effectively integrated to answer the research question?** | **3. Are the outputs of the integration of qualitative and quantitative components adequately interpreted?** | **4. Are divergences and inconsistencies between quantitative and qualitative results adequately addressed?** | **5. Do the different components of the study adhere to the quality criteria of each tradition of the methods involved?** |
| Hospital Strategies for Reducing Emergency Department Crowding: A Mixed-Methods Study. (Chang et al., 2018) | Yes | Yes | Yes | Yes | Yes | Yes | Yes |
| **Quality Appraisal N/A** |  |  |  |  |  |  |  |
| Combating infections at Maine Medical Center: Insights into complexity-informed leadership from positive deviance. (Lindberg & Schneider, 2013) | No |  |  |  |  |  |  |
| Methicillin-resistant Staphylococcus aureus (MRSA) prevention through facility-wide culture change. (Bonuel et al., 2009) | No |  |  |  |  |  |  |
| Creating a culture of innovation in nursing education through shared vision, leadership, interdisciplinary partnerships, and positive deviance. (Melnyk & Davidson, 2009) | No |  |  |  |  |  |  |
| Reducing Infections “Together”: A review of Socioadaptive Approaches. (Sreeramoju, 2019) | No |  |  |  |  |  |  |
| People, systems and safety: resilience and excellence in healthcare practice. (Smith & Plunkett, 2019) | No |  |  |  |  |  |  |
| Positive Deviance: A New Tool for Infection Prevention and Patient Safety. (Marra et al., 2013) | No |  |  |  |  |  |  |
| Exploring the concept and use of positive deviance in nursing. (Gary, 2013) | No |  |  |  |  |  |  |
| Positive deviance: An elegant solution to a complex problem. (Lindberg & Clancy, 2010) | No |  |  |  |  |  |  |
| Diamonds in the rough: positive deviance and complexity. (Clancy, 2010) | No |  |  |  |  |  |  |
| Positive deviance: a different approach to achieving patient safety. (Lawton et al., 2014) | No |  |  |  |  |  |  |
| Positive deviance: innovation from the inside out. (Jaramillo et al., 2008) | No |  |  |  |  |  |  |
